# Acute L-Arginine supplementation does not increase nitric oxide production in healthy subjects

**DOI:** 10.1186/1743-7075-9-54

**Published:** 2012-06-12

**Authors:** Thiago Silveira Alvares, Carlos Adam Conte-Junior, Joab Trajano Silva, Vânia Margaret Flosi Paschoalin

**Affiliations:** 1 Laboratory of Advanced Analysis in Biochemistry and Molecular Biology, Department of Biochemistry, Chemistry Institute, Federal University of Rio de Janeiro, Brazil; 2 Present address: Laboratório de Análises Avançadas em Bioquímica e Biologia Molecular, Departamento de Bioquímica – Instituto de Química, Universidade Federal do Rio de Janeiro, Avenida Athos da Silveira Ramos, 149 Centro de Tecnologia, Bloco A, Sala 545, Ilha do Fundão, Rio de Janeiro, RJ, 21941-909, Brazil

**Keywords:** Amino acids, Nitric oxide, Asymmetric dimethylarginine, Symmetric dimethylarginine, Nitrite, Nitrate, HPLC

## Abstract

Dietary supplements containing L-arginine have been marketed with the purpose of increasing vasodilatation, and thus, blood and oxygen supply to the exercising muscle. The present study evaluated the acute effect of L-arginine supplementation on indicators of NO production, nitrite (NO_2_^-^) + nitrate (NO_3_^-^) (NOx), in healthy subjects. Plasma concentrations of asymmetric dimethylarginine (ADMA) and symmetric dimethylarginine (SDMA) have also been addressed. Seventeen healthy males participated in a randomized, double-blind, placebo-controlled study. Blood samples were drawn from a left antecubital vein at baseline (T0). Afterwards, subjects were randomly submittedto 6 g of oral L-arginine supplementation (as L-arginine hydrochloride) or placebo (as corn starch); afterwards, the subjects remained at rest in supine position and blood samples were drawn again at 30 (T1), 60 (T2), 90 (T3) and 120 minutes (T4) after supplementation. To analyze NO production, NO_3_^-^ was converted to NO_2_^-^ by nitrate reductase, followed by the derivatization of NO_2_^-^ with 2,3-diaminonaphthalene. NOx, ADMA and SDMA were analyzed using a high-performance liquid chromatography system and monitored with a fluorescence detector. Two-way ANOVA with repeated measures showed no significant changes in NOx concentrations on the L-arginine group as compared to placebo group at any of the fivetime points (T0: 17.6 ± 3.9 vs 14.6 ± 2.3 μmol/L; T1: 15.8 ± 2.4 vs 14.3 ± 1.7 μmol/L; T2: 16.8 ± 4.9 vs 13.7 ± 2.7 μmol/L; T3: 16.7 ± 3.9 vs 14.6 ± 2.1 μmol/L; T4: 15.1 ± 2.8 vs 13.5 ± 3.5 μmol/L). Furthermore, plasma levels of ADMA and SDMA were not statistically significant between the L-arginine and placebo groups at T0 (0.43 ± 0.19 vs 0.39 ± 0.15 μmol/L and 1.83 ± 1.13 vs 1.70 ± 0.62 μmol/L), respectively. In conclusion, acute L-arginine supplementation does not increase plasma concentration of NOx in healthy individuals with normal plasma concentrations of ADMA.

## Introduction

Many supplements have been introduced in the market with the purpose of enhancing athletes’ performance [1]. Most of these supplements allegedly help athletes tolerate a higher degree of heavy training by helping athletes recover faster during intense sport training [2]. Recently, supplements containing L-arginine have been introduced in the market claiming to promote vasodilatation by increasing nitric oxide (NO) production via nitric oxide synthase (NOS) activation. This vasodilatation would favor an increase perfusion as well as a higher nutrient and oxygen delivery to the active muscles during exercise, enhancing protein synthesis and muscle recovery [2].

L-arginine is considered a semi-essential amino acid because the body normally produces it in sufficient amounts. However, supplementation may be needed in special conditions such as malnutrition, excessive ammonia production, burns, infections, peritoneal dialysis, rapid growth, urea synthesis disorders, and/or sepsis [3].

Physiological concentrations of L-arginine in healthy individuals are enough to saturate endothelial NOS, which is ~ 3 μmol/L. Therefore, supplementary L-arginine should not promote increased enzyme activity; consequently, no further NO production should occur. However, there is evidence describing the NO-mediated biological effects associated with L-arginine supplementation despite the fact that nitric oxide synthase (NOS) is theoretically saturated with the physiological concentration of L-arginine—hence the condition known as the ‘L-arginine paradox’ [4].

Early evidence suggests that L-arginine supplementation may help treat individuals with atherosclerosis risk factors, such as hypercholesterolemia, hypertension, diabetes mellitus, kidney failure, hyperhomocysteinemia, smoking, and aging—all of which are conditions that are associated with reduced NO biosynthesis [5-9]. Böger R., [10] had shown that plasma levels of asymmetric dimethylarginine (ADMA), an endogenous NOS inhibitor, are increased approximately 2–3 fold in the pathophysiological conditions associated with cardiovascular disease. For this reason, elevated ADMA concentration may be one possible explanation for endothelial dysfunction and decreased NO synthesis in this disease cluster. Therefore, it appears that only subjects with poor NO synthesis are likely to benefit from L-arginine supplementation.

Despite the theory regarding L-arginine supplementation improving vasodilatation from increased NO production, a recent review [2] about the ergogenic effect of L-arginine supplementation in healthy subjects shows that there were only five studies that evaluated exercise performance after acute L-arginine supplementation, three of which reported significant improvements. Besides the improvements observed in physical performance, the authors of these studies did not measure the underlying mechanism that could explain how the results obtained may have been due to increased NO production.

Bailey et al., [11] observed significant increases in the time to task failure with concomitant reductions in the O2 cost of moderate-intensity cycle exercise and slow oxygen uptake component amplitude on the group supplemented with 6 g of L-arginine, 1 h before a series of moderate- and severe-intensity exercise bouts for 3 days. Stevens et al., [12] observed significant increase in peak torque, total work and fatigue index after supplementing with a product containing 6 g of L-arginine in three equal aliquots at 45, 30 and 10-min periods before isokinetic dynamometer exercise. Buford and Koch, [13] observed significant improvement of average power during repeated sets of supra-maximal exercise during cycle ergometer on the group that consumed 6 g of L-arginine.

Based on the theory that physiological concentrations of L-arginine are enough to saturate endothelial NOS and no further NO production should occur in healthy individuals, it is our hypothesis that there should be no change inplasma concentration of NO_2_^-^ and NO_3_^-^ as a result of L-arginine supplementation when compared to placebo.

Due to the fact that other studies have demonstrated vascular and exercise performance benefits after L-arginine supplementation in healthy individuals [2,14], the question still remains as to whether this effect is NO-mediated. Therefore, in order to test the claim that L-arginine supplementation may increase NO synthesis, the present study was conducted to identify the acute effects of L-arginine supplementation on indirect markers of NO synthesis—NO_2_^-^ and NO_3_^-^. It is to point out that, contrary to the present study, other studiesthat evaluated the effect of L-arginine supplementation on NO synthesis at rest have methodological limitations [15] (e.g.: They did not control diet for food contain NO_2_^-^ and NO_3_^-^), which may cause flaws in the results. The plasma levels of ADMA and SDMA at the beginning of the study have also been addressed.

## Methods

### Subjects

Seventeen healthy males (25.5 ± 3.5 yr, 78.7 ± 10.5 kg; 176.1 ± 7.5 cm and 25.3 ± 2.3 kg.m^-2^ BMI) were recruited to participate in the study. All subjects were fully informed of the nature and purpose of the investigation and gave their written consent to participate. The exclusion criteria for participation in the study were any known cardiovascular, pulmonary or metabolic diseases (asthma, diabetes, hypertension, dyslipidemia etc.), and the use of nutritional and pharmacological ergogenics. All experimental procedures were performed in accordance with the ethical standards of the Helsinki Declaration and were approved by the Institutional Ethics Committee of the Hospital Universitário Clementino Fraga Filho (protocol # 0118.0.197.000-10) from Rio de Janeiro, Brazil.

### Experimental design

**Figure 1:**
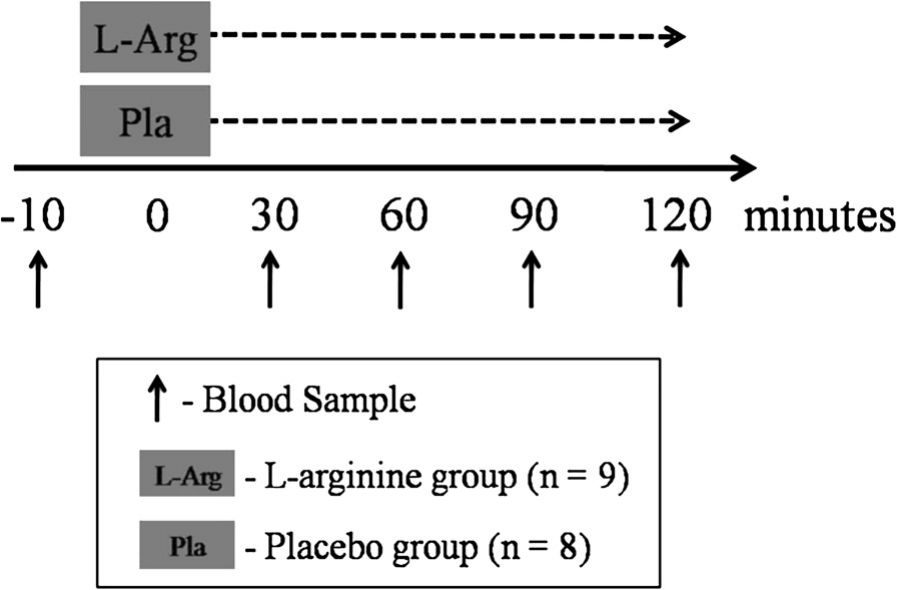
Summary of experimental design.

In a randomized, double-blind and placebo-controlled study, subjects reported to the laboratory. Blood samples were drawn from an antecubital forearm vein at baseline after a ten-minute period of quiet rest in supine position. Afterwards, subjects were randomly divided into either a placebo or an L-arginine group and rested again in supine position in a quiet room. Blood samples were drawn again at 30 (T0), 60 (T1), 90 (T2) and 120 minutes (T4) after supplementation (see Figure 1). During this period, the subjects consumed no food and drink.

### Dietary control

One day before conducting the study, the subjects were oriented as to the NO_2_^-^ and NO_3_^-^ content of foods and were requested to restrict their diets from foods rich in NO_2_^-^ and NO_3_^-^. A list describing foods and groups of food to be avoided and to be preferred was distributed to the subjects, in order to simplify their dietary choices for low NO_2_^-^ and NO_3_^-^ foods for the 24-hour period prior to the study. In short, the subjects were advised to avoid vegetable products, such as spinach and squash, which contain the highest amounts of NO_3_^-^ per serving. Sweets, nuts, fats and oils contain very little NO_3_^-^ per serving and were thus permitted. Red meat (beef, pork, lamb, mutton, and liver) and bean products contain the highest amounts of dietary NO_2_^-^ per serving and were to be avoided. Negligible sources of dietary NO_2_^-^ are found in cottage cheese, fats such as butter or margarine, and various fruit juices. This dietary orientation was based on a list developed to estimates of dietary NO_2_^-^ and NO_3_^-^[16]. Adherence to the diet was controlled by twenty-four-hour recall conducted upon arrival for the study, in which each subject was interrogated as to their dietary intake for the 24 h period prior to arrival for the study. Further analysis of energy intake and macronutrients (as a percentage of total energy) from the twenty-four-hour recall of each subject of both L-arginine and placebo groups was performed by using a nutrition analysis software program (dietWin Professional 2008 for windows, version 2.0, Porto Alegre, RS, Brazil).

### Supplementation

Ten minutes after baseline blood sample, all subjects were orally administered either 6 g of encapsulated L-arginine hydrochloride or placebo (as corn starch) in identical forms with 400 mL of H_2_O Milli-Q in a double-blind and randomized manner. We chose to provide 6 g of L-arginine, because such a dose would be well-tolerated when consumed orally, and was reported to increase vasodilatation [17].

### Nitric oxide production

The blood was drawn from antecubital veins and collected in EDTA-containing tubes, and then immediately centrifuged at 3000 *g* for 10 min at 4°C in order to separate the plasma, before storing it at −80°C for later analysis. NO production was assayed by measuring plasma NO_2_^-^ + NO_3_^-^ (NOx) as previously described by Li et al. [18]. In brief, plasma was diluted in a proportion of 1:10 and 1:100 in order to analyze NO_2_^-^ and NO_3_^-^, respectively. After dilution, 1 mL of each sample were filtered using a 10-kDa cutoff ultrafilter (Vivaspin 2, GE Healthcare®) at 14000 *g* for 15 min to remove high-molecular weight proteins. NO_3_^-^ was converted to NO_2_^-^ enzymatically by nitrate reductase EC 1.6.6.2 (Roche Diagnostics, Mannheim, Germany) from *Aspergillus species*. The solution, which consisted of 200 μL of sample, 120 μM NADPH and 2 μM FAD, was incubated at room temperature for 1 h. Following the conversion of NO_3_^-^ to NO_2_^-^, the sample was incubated at 24°C with 316 mM 2,3-diaminonaphthalene to convert NO_2_^-^ into the highly fluorescent 2,3-naphthotriazole followed by addition of 2.8 M NaOH and immediately analyzed by high-performance liquid chromatography (HPLC). The HPLC device was equipped with a 5-μm reversed-phase C8 column Discovery® (150 x 4,6 mm, I.D.) guarded by a 40-μm reversed-phase C18 guard column Ascentis® (50 x 4,6 mm, I.D.) and a fluorescence detector model RF-10AXL (Shimadzu®) monitoring excitation and emission wavelengths at 375 nm and 415 nm, respectively. Both columns were obtained from Supelco (Bellefonte, PA, USA). The mobile phase (1.0 mL/min) was 15 mM sodium phosphate buffer (pH 7.5) and methanol.

Oxidation of NO via several metabolic reactions results in the formation of nitrite (NO_2_^-^) and nitrate (NO_3_^-^) as the two major end products [19]. The principal oxidation product of NO synthesis in aqueous solutions (in the absence of biological constituents such as hemoproteins) is NO_2_^-^. The further oxidation to NO_3_^-^ requires the presence of additional oxidizing species such as oxyhemoproteins [20]. For example, NO is quickly oxidized to NO_2_^-^ via autoxidation in aqueous solutions, such as biological fluids, and may react with superoxide anions to produce peroxynitrites. In the presence of heme groups in proteins such as hemoglobin and myoglobin, NO reacts with oxyhemoglobin to produce metahemoglobin and NO_3_^-^. Therefore, measurement of NO_2_^-^ and NO_3_^-^ in various biological fluids turned out to be the most suitable, practical and reliable non-invasive method to assess systemic NO synthesis in vivo [19].

### Plasma amino acids

The amino acids L-arginine, L-citrulline and L-ornithine were analyzed as previously described by Wu and Meininger [21]. In brief, 50 μL of plasma was mixed with 50 μL of 1.5 M perchloric acid (v/v) to remove proteins. After 2 min at room temperature, 1.125 mL H_2_O and 25 μL potassium carbonate was added. The tubes were centrifuged at 10000 *g* for 2 min. The sample (25 μL) was mixed with 25 μL of the o-phthaldialdehyde (OPA) reagent solution (v/v) for 1 min. The solution derivatized was immediately analyzed by HPLC. The HPLC device was equipped with a 3-μm reversed-phase C18 column Kromasil® (150 x 4.6 mm, I.D.) guarded by a 40-μm reversed-phase C18 guard column Ascentis® (50 x 4.6 mm, I.D.) and a fluorescence detector model RF-10AXL (Shimadzu®) monitoring excitation and emission wavelengths at 340 nm and 455 nm, respectively. These chromatographic methods are highly sensitive, specific, and accurate, as well as provide a useful tool to study the L-arginine—NO pathway.

### ADMA and SDMA analysis

The plasma concentrations of ADMA and SDMA were analyzed as previously described by Wu and Meininger [21]. In brief, 200 μL of plasma was mixed with 100 μL of 1.5 M perchloric acid (v/v) to remove proteins, followed by 50 μL of 2 M potassium carbonate and 700 μL of phosphate buffer (pH 7.0). The whole solution was loaded into a solid-phase extraction column (Oasis MCX) and the elution solvent was removed using a sample concentrator system (Savant SpeedVac Concentrator, Thermo Fisher Scientific Inc.). The residues were suspended in 200 μL H_2_O. The sample (15 μL) was mixed with 15 μL of the OPA reagent (v/v) for 1 min. The solution derivatized was immediately analyzed by HPLC. The HPLC device was equipped with a Nucleosil 100–5 C6H5 column (250 x 4.6 mm, I.D; Manchery Nagel, Easton, PA) and a fluorescence detector model RF-10AXL (Shimadzu®) monitored excitation and emission wavelengths at 340 nm and 455 nm, respectively. All chromatographic procedures were performed at room temperature.

## Statistical analysis

A Two-way ANOVA with repeated measures on two factors (2 x 5; group x time) was utilized to identify differences in NOx and plasma amino acids at each time point. Calculation of the integrated plasma NOx concentration [area under the curve (AUC)] was determined by the use of a trapezoidal method (baseline NOx concentration: y = 0). Unpaired Student *t*-test was utilized to identify differences in plasma concentrations of ADMA, SDMA and L-arginine/ADMA ratio at the onset of the study. Statistical significance was set at the 0.05 level of confidence. All analyses were performed using GraphPad Prism version 5.00 for Windows (GraphPad Software, San Diego California USA).

## Results

### Subject characteristics

At the study onset there were no significant differences between the randomly assigned placebo versus L-arginine groups with respect to age, height, body weight, BMI, body fat (see Table 1).

**Table 1:** Subject’s baseline characteristics

	**L-Arg (N = 9)**	**Pla (N = 8)**
Age (yr)	26.0 ± 4.6	24.9 ± 1.7
Height (cm)	175.4 ± 7.7	177.0 ± 7.6
Body weight (kg)	79.3 ± 12.5	78.1 ± 8.4
BMI (kg.m^-2^)	25.7 ± 2.4	24.9 ± 2.3
Body fat (%)	14.4 ± 5.6	16.4 ± 2.5
ADMA (μmol/L)	0.43 ± 0.19	0.39 ± 0.15
L-arginine/ADMA ratio	453 ± 282	438 ± 272
SDMA (μmol/L)	1.83 ± 1.13	1.70 ± 0.62

The values are mean ± standard deviation. L-Arg = L-arginine supplemented group; Pla = Placebo group.

### Dietary control

Based on the evaluation of the twenty-four-hour recall, all subjects of both the L-arginine and placebo groups had apparently adhered to the dietary orientation and avoided all foods listed as high in NO_2_^-^ and NO_3_^-^. There was no significant difference in energy intake and percentage of macronutrients before 24 h of beginning the study between L-arginine and placebo groups (2663 ± 508.5 vs 2905 ± 641.6 kcal; 52.9 ± 6.3 vs 53.6 ± 5.8% carbohydrates; 21.6 ± 4.8 vs 21.7 ± 5.1% protein; 25.5 ± 7.9 vs 24.7 ± 6.4% fat).

### Nitric oxide production

**Figure 2:**
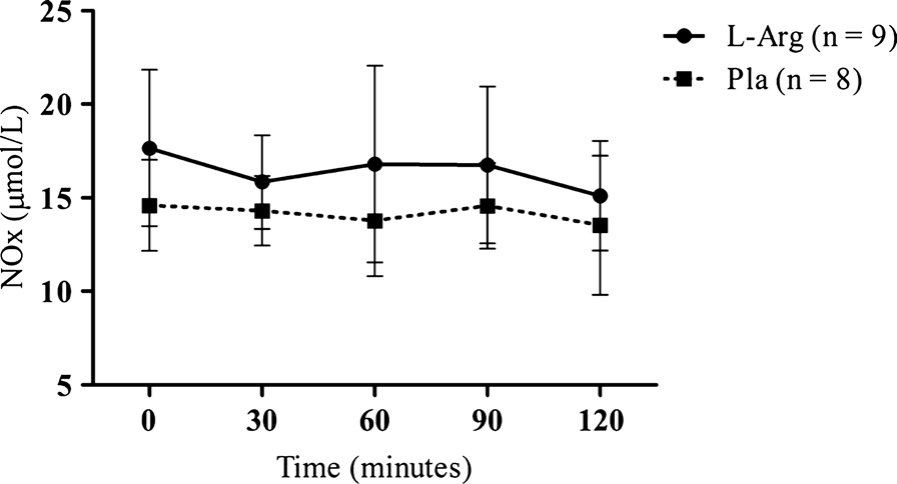
**Plasma NOx concentrations (μmol/L).** No significant change was observed between groups at any time point. L-Arg = L-arginine supplemented group; Pla = Placebo group.

**Figure 3:**
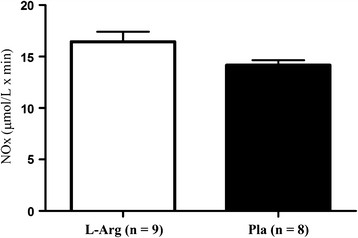
**Total integrated NOx area under curve during the 120 min post supplementation period.** No significant change was observed between groups over the time. L-Arg = L-arginine supplemented group; Pla = Placebo group.

Plasma NO_2_^-^ + NO_3_^-^ (NOx) concentrations at each time point are depicted in Figure 2. No significant differences were found between groups at any time point. Figure 3 presents the data for the total integrated NOx AUC during the 120 min post supplementation period. The integrated AUC revealed that despite a 15% higher, the L-arginine supplementation did not resulted in greater NOx response than placebo group over the time (*P* > 0.05).

### Plasma amino acids

**Figure 4:**
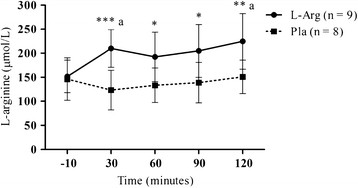
**Plasma concentration of L-arginine. ** The symbols ***(*P* < 0.001), **(*P* < 0.01) and *(*P* < 0.05) denotes significantly different from placebo; ^a^(*P* < 0.01) denotes significantly different from 0 minutes. L-Arg = L-arginine supplemented group; Pla = Placebo group.

The plasma concentrations of L-arginine, L-citrulline and L-ornithine at each time point are depicted in Table 2. No significant difference between groups was observed at baseline. Plasma L-arginine increased significantly at 30 min (209.9 ± 39.1 vs 123.3 ± 44.6 μmol/L, *P* < 0.001), 60 min (192.3 ± 51.3 vs 133.4 ± 36.0 μmol/L, *P* < 0.05), 90 min (204.9 ± 55.1 vs 138.8 ± 42.2 μmol/L, *P* < 0.05) and 120 min (224.7 ± 57.8 vs 150.8 ± 34.8 μmol/L, *P* < 0.01) after supplementation in the L-arginine group, when compared to the placebo group (see Figure 4). No significant change was observed between groups in plasma L-citrulline and L-ornithine at any time point.

**Table 2:** Values of plasma amino acids (μmol/L) at −10, 30, 60, 90 and 120 minutes post-supplementation

**Amino acid**	**L-Arg (n =9)**					**Pla (n =8)**				
	**−10**	**30**	**60**	**90**	**120**	**−10**	**30**	**60**	**90**	**120**
L-citrulline	57.4	125.2	75.2	89.7	82.2	56.2	76.9	66.2	75.9	79.8
	±31.2	±136.3	±45.4	±92.9	±78.0	±28.1	±26.3	±43.0	±53.9	±63.0
L-arginine	151.7	209.9	192.3	204.9	224.7	146.2	123.3	133.4	138.8	150.8
	±33.9	±39.1^*** †^	±51.3^*^	±55.1^*^	±57.8^* a^	±44.3	±44.6	±36.0	±42.2	±34.8
L-ornithine	127.5	153.2	154.0	143.1	147.0	93.4	119.8	101.1	105.5	110.4
	±87.1	±84.9	±65.7	±55.9	±65.4	±23.7	±88.8	±49.6	±59.9	±59.2

The values are mean ± standard deviation. The symbols ^***^(*P* < 0.001), ^**^(*P* < 0.01) and ^*^(*P* < 0.05) denotes significantly different from placebo at same time point; ^a^ denotes significantly different from time −10. L-Arg = L-arginine supplemented group; Pla = Placebo group.

### Plasma ADMA and SDMA

At the onset of the study, there were no significant differences in plasma levels of ADMA, SDMA and L-arginine/ADMA ratio between the randomly assigned placebo versus L-arginine groups (0.43 ± 0.19 vs 0.39 ± 0.15 μmol/L and 1.83 ± 1.13 vs 1.70 ± 0.62 μmol/L) (see Table 1).

## Discussion

Dietary supplements containing the semi-essential amino acid L-arginine (the only substrate of NOS) have been introduced in the market, claiming to promote vasodilatation by increasing production of NO. In the present study, we found that in healthy subjects 6 g of oral L-arginine supplementation did not stimulate an increase in NO production when compared with the placebo group. Additionally, no significant increases were observed in plasma L-citrulline, which is the by-product of NO synthesis from L-arginine. Furthermore, no significant differences in plasma concentrations of ADMA and SDMA at onset of the study were observed between the L-arginine and placebo groups.

Several studies had showed no significant difference in NO production (measured by NO_2_^-^ and NO_3_^-^) after L-arginine supplementation [22-24]. Liu et al. [22] did not observe any significant differences in plasma NO_2_^-^ and NO_3_^-^ concentrations after orally supplementing ten healthy male athletes with 6 g of L-arginine (as free form) or placebo for 3 days. Koppo et al. [23] observed no significant difference in urinary NO_2_^-^ and NO_3_^-^ after 14 days of supplementing seven physically active healthy males with 7.2 g of L-arginine hydrochloride (3 × 3 capsules of 805 mg), and Tang et al. [24] also did not observed any significant difference on NO synthesis (measured by plasma NO_2_^-^ and NO_3_^-^) in eight healthy young men after an single dose of 10 g of L-arginine. Since all subjects of these studies were submitted to exercise, the results of these studies are not surprising due to the very nature of the underlying mechanism of NO synthesis: vascular shear stress is considered the main stimulus for endothelial NO production during exercise [25]. Therefore, there should theoretically be no need for supplementary L-arginine to synthesize NO during exercise.

Regarding studies at rest condition, Blum et al. [26] investigated the effects of oral L-arginine (9 g daily for one month) on NO bioactivity in 10 healthy postmenopausal women. After the supplementation period, the authors observed no significant difference in serum NO_2_^-^ and NO_3_^-^ concentrations. In another study, Evans et al. [27] also did not find significant differences in serum NO_2_^-^ and NO_3_^-^ concentrations after submitting twelve healthy subjects to take L-arginine for 1-week periods at daily doses of 3, 9, 21, and 30 g. Schwedhelm et al., [28] published a study with the purpose of investigating the pharmacokinetic and pharmacodynamics properties of oral L-arginine with regard to NO metabolism. Twenty healthy subjects were submitted to two doses of L-arginine supplementation (1 g of arginine sustained-release tree times per day or 1.6 g of immediate-release arginine two times per day) for 7 days. At baseline and on day 7, a single dose of L-arginine supplementation (half of the total daily dose, respectively) was administered and blood samples were drawn at 0, 0.5, 1, 2, 3, 4, 6, 8, 12, 16 and 24 h after supplementation. The authors observed no significant differences in urinary excretion of nitrate at any time point, despite increases in plasma L-arginine concentrations after the supplementation (T_max_ = 3.7 ± 1.3 h for arginine sustained-release and 0.7 ± 0.1 h for arginine immediate-release). In the present study, the plasma L-arginine concentrations increased significantly after 30 min of L-arginine supplementation when compared to placebo group, and maintained high throughout the study period. Furthermore, no significant differences were observed in plasma concentrations of L-ornithine and L-citrulline at any time point between the groups.

It is important to point out that, contrary to the present study, neither of the above mentioned studies controlled the intake of food rich in NO_2_^-^ and NO_3_^-^ from subject’s diet before or during the study. Most NO_2_^-^ and NO_3_^-^ comes from diet (vegetable products contain the highest levels of NO_3_^-^; meat and bean products contain the highest levels of NO_2_^-^), which may alter the results of the analysis. Thus, endogenous synthesis of NO may not be adequately measured by NO_2_^-^ and NO_3_^-^ in plasma and urine if the diet is not controlled.

Measurement of NO_2_^-^ and NO_3_^-^ in various biological fluids turned out to be the most suitable, practical and reliable non-invasive method to assess systemic NO synthesis in vivo [19]. In the present study, NO synthesis was quantified by measuring plasma NO_2_^-^ + NO_3_^-^ (NOx) via high-performance liquid chromatography. Most of the studies [22-24] had analyzed NO synthesis by using Griess reaction. The acidic conditions under which experiments using Griess reaction are conducted favor the formation of S-nitroso compounds from NO_2_^-^ and reduced thiols. Thus, the measurement of NO_2_^-^ and NO_3_^-^ in biological fluids by assays based on the Griess is subject to interference by many substances acting at different places in the Griess reaction and the following spectrophotometric measurement in the same wavelength (normal absorbance at 540 nm). Therefore, studies using Griess reaction to detect stable metabolites (NO_2_^-^ and NO_3_^-^) to measure NO synthesis may have significant methodological limitations, as compared to fluorometric techniques associated with HPLC.

Bailey et al. [11] observed significant increases in plasma NO_2_^-^ after supplementing nine healthy recreationally active men with a supplement that contained 6 g of L-arginine (dissolved in 500 mL of water) as compared to placebo. It is important to note that this study associated other amino acids besides L-arginine, including L-citrulline (quantities not expressed in the study), which have been shown to increase NO production, as measured by plasma concentrations of NO_2_^-^[29] and urinary excretion of NO_3_^-^ and cGMP [28]. Interestingly, the authors did not measure plasma NO_2_^-^ at baseline; they had just done so 1 hour after supplementation, which is a major methodological limitation, since it is not known whether there were any differences in the samples prior to supplementation. Furthermore, taking into consideration that diet can influence nitrite plasma concentrations, no dietary control to limit the consumption of foods rich in NO_2_^-^ and NO_3_^-^ was conducted.

Others studies also have showed improvements in NO production by using L-arginine supplementation [30-33]. However, all of these studies had administered L-arginine in subjects with some cardiovascular risk factors or cardiopathy. It appears that L-arginine is a limiting factor for NO synthesis in patients at risk for atherosclerosis, but not for healthy individuals [34]. Therefore, L-arginine supplementation may be necessary only for individuals with atherosclerosis risk factors.

Among the possible explanations for this phenomenon is the presence of high levels of asymmetric dimethylarginine (ADMA), an endogenous NOS inhibitor. Higher concentrations of ADMA were encountered in individuals with atherosclerosis, as well as in individuals with atherosclerosis risk factors, such as hypercholesterolaemia, hypertension, diabetes mellitus, kidney failure, hyperhomocysteinaemia, smoking and aging [10]. Physiological levels of L-arginine and the presence of normal concentrations of ADMA saturate the endothelial NOS enzyme, promoting NO production. In these conditions, L-arginine supplementation does not affect enzyme activity. In contrast, in the presence of elevated plasma concentrations of ADMA the endothelial NOS activity diminishes, resulting in lower physiological levels of NO production. SDMA has no effect on NOS activity but may compete with L-arginine for the y + transport system [35]. Under these conditions, L-arginine supplementation may re-establish the L-arginine/ADMA ratio in order to activate endothelial NOS [4]. Therefore, L-arginine supplementation may exert a beneficial effect on vascular function.

In the present study, we observed no significant difference in plasma concentrations of ADMA, SDMA and L-arginine/ADMA ratio at baseline between the groups. This finding may explain the absence of significant changes in NO production after L-arginine supplementation. The baseline plasma concentrations of ADMA observed in the present study are similar to previous studies in healthy subjects which reported ADMA concentrations ranging between 0.3 and 0.9 μmol/L [36-38]. Therefore, it may be speculated that there should be no further increase in NO production after L-arginine supplementation in subjects with normal levels of ADMA.

Böger et al., [34] observed that plasma ADMA levels were significantly higher in hypercholesterolemic subjects than in normocholesterolemic control subjects (2.17 ± 0.15 and 1.03 ± 0.09 μmol/L, respectively); and the higher levels of ADMA was associated with reduced NO synthesis. Therefore, it appears that high plasma ADMA concentrations may inhibit NOS enzyme, but low or normal plasma ADMA concentrations do not affect NO synthesis.

In conclusion, L-arginine supplementation does not increase NO production in healthy subjects with normal plasma ADMA concentrations. Therefore, it is not advisable to recommend dietary supplements containing L-arginine for the purposes of increasingacutely NO production in healthy subjects. This result does not discard the possible effect of L-arginine on NO production in individuals with pathophysiologicalconditions (e.g.: hypercholesterolaemia, hypertension, diabetes mellitus, kidney failure, hyperhomocysteinaemia, smoking and aging) and long-term studies are needed to identify whether L-arginine may provide some benefit.

## Abbreviations

NO, Nitric oxide; NOS, Nitric oxide synthase; NOx, Nitrite + nitrate; NO_2_^-^, Nitrite; NO_3_^-^, Nitrate; HPLC, High-performance liquid chromatography; ANOVA, Analysis of variance; ADMA, Asymmetric dimethylarginine; SDMA, Symmetric dimethylarginine.

## Competing interests

Each author certifies that he or she has no commercial associations (e.g., consultancies, stock ownership, equity interest, patent/licensing arrangements, etc.) that might pose a conflict of interest in connection with the submitted article, except as disclosed on a separate attachment. All funding sources supporting the Work and all institutional or corporate affiliations of the authors are acknowledged in a footnote in the Work.

## Authors’ contributions

TSA contributed substantially to data acquisition and chromatographic analysis, statistical analysis and data interpretation, and was the manuscript writer. CACJ contributed substantially to chromatographic analysis, interpretation of results, and reviewing the manuscript. JTS contributed to data interpretation and manuscript revision. VMFP contributed to data interpretation and manuscript revision. All authors read and approved the final manuscript.

## References

[B1] Kreider RB Wilborn CD Taylor L Campbell B Almada AL Collins R Cooke M Earnest CP Greenwood M Kalman DS Kerksick CM Kleiner SM Leutholtz B Lopez H Lowery LM Mendel R Smith A Spano M Wildman R Willoughby DS Ziegenfuss TN Antonio J ISSN Exercise & Sport Nutrition Review: Research & Recommendations J Int Soc Sports Nutr 2010 7 7 10.1186/1550-2783-7-720181066

[B2] Alvares TS Meirelles CM Bhambhani YN Paschoalin VM Gomes PS L-arginine as a Potential Ergogenic Aid in Healthy Subjects Sports Med 2011 41 3 233 248 10.2165/11538590-000000000-00000 21395365

[B3] Appleton J Arginine: Clinical potential of a semi-essential amino Altern Med Rev 2002 7 6 512 522 12495375

[B4] Bode-Böger SM Scalera F Ignarro LJ The L-arginine paradox: importance of the L-arginine/asymmetrical dimethylarginine ratio PharmacolTher 2007 114 3 295 306 10.1016/j.pharmthera.2007.03.002 17482266

[B5] Creager M Gallagher S Girerd X Coleman S Dzau V Cooke J L-Arginine improves endothelium-dependent vasodilation in hypercholesterolemic humans J Clin Invest 1992 90 4 1248 1253 10.1172/JCI115987 1401062 PMC443166

[B6] Clarkson P Adams M Powe A Donald A McCredie R Robinson J McCarthy S Keech A Celermajer D Deanfiel J Oral L-Arginnine improves endothelium-dependent dilation in hypercholesterolemic young adults J Clin Invest 1996 97 8 1989 1994 10.1172/JCI118632 8621785 PMC507270

[B7] Pieper G Siebeneich W Dondlinger L Short-term oral administration of L-arginine reverses defective endothelium-dependent relaxation and cGMP generation in diabetes Eur J Pharmacol 1996 317 2–3 317 320 8997616 10.1016/s0014-2999(96)00831-x

[B8] Adams M McCredie R Jessup W Robinson J Sullivan D Celermajer D Oral L-Arginine improves endothelium-dependent dilatation and reduces monocyte adhesion to endothelial cells in young men with coronary artery disease Atherosclerosis 1997 129 2 261 269 10.1016/S0021-9150(96)06044-3 9105569

[B9] Lerman A Burnett J Higano S McKinley L Holmes D Long-term L-Arginine supplementation improves small-vessel coronary endothelial function in humans Circulation 1998 97 21 2123 2128 10.1161/01.CIR.97.21.2123 9626172

[B10] Böger R Asymmetric Dimethylarginine, an Endogenous Inhibitor of Nitric Oxide Synthase, Explains the “L-Arginine Paradox” and Acts as a Novel Cardiovascular Risk Factor J Nutr 2004 134 10 2842S 2847S 15465797 10.1093/jn/134.10.2842S

[B11] Bailey SJ Winyard PG Vanhatalo A Blackwell JR DiMenna FJ Wilkerson DP Jones AM Acute L-arginine supplementation reduces the O2 cost of moderate-intensity exercise and enhances high-intensity exercise tolerance J Appl Physiol 2010 109 5 1394 1403 10.1152/japplphysiol.00503.2010 20724562

[B12] Stevens BR Godfrey MD Kaminski TW Braith RW High-intensity dynamic human muscle performance enhanced by a metabolic intervention Med Sci Sports Exerc 2000 32 12 2102 2108 10.1097/00005768-200012000-00021 11128858

[B13] Buford BN Koch AJ Glycine-arginine-alpha-ketoisocaproic acid improves performance of repeated cycling sprints Med Sci Sports Exerc 2004 36 4 583 587 10.1249/01.MSS.0000122075.14060.C4 15064584

[B14] Bode-Böger SM Muke J Surdacki A Brabant G Böger RH Frölich JC Oral L-arginine improves endothelial function in healthy individuals older than 70 years Vasc Med 2003 8 2 77 81 10.1191/1358863x03vm474oa 14518608

[B15] Forbes SC Bell GJ The acute effects of a low and high dose of oral L-arginine supplementation in young active males at rest Appl Physiol Nutr Metab 2011 36 3 405 411 10.1139/h11-035 21574873

[B16] Griesenbeck JS Steck MD Huber JC Development of estimates of dietary nitrates, nitrites, and nitrosamines for use with the short Willet food frequency questionnaire Nutr J 2009 6 8 16 10.1186/1475-2891-8-16 PMC2669451 19348679

[B17] Bode-Böger SM Böger RH Galland A Tsikas D Frölich JC L-arginine-induced vasodilation in healthy humans: pharmacokinetic-pharmacodynamic relationship Br J Clin Pharmacol 1998 46 5 489 497 9833603 10.1046/j.1365-2125.1998.00803.x PMC1873701

[B18] Li H Meininger CJ Wu G Rapid determination of nitrite by reversed-phase high-performance liquid chromatography with fluorescence detection J Chromatogr B Biomed Sci Appl 2000 746 2 199 207 10.1016/S0378-4347(00)00328-5 11076072

[B19] Tsikas D Methods of quantitative analysis of the nitric oxide metabolites nitrite and nitrate in human biological fluids Free Radic Res 2005 39 8 797 815 10.1080/10715760500053651 16036360

[B20] Ignarro LJ Fukuto JM Griscavage JM Rogers NE Byrns RE Oxidation of nitric oxide in aqueous solution to nitrite but not nitrate: Comparison with enzymatically formed nitric oxide from L-arginine Proc Natl Acad Sci USA 1993 90 17 8103 8107 10.1073/pnas.90.17.8103 7690141 PMC47296

[B21] Wu G Meininger CJ Analysis of Citrulline, Arginine, and Methylarginines using High-Performance Liquid Chromatography Methods Enzymol 2008 440 177 189 18423217 10.1016/S0076-6879(07)00810-5

[B22] Liu TH Wu CL Chiang CW Lo YW Tseng HF Chang CK No effect of short-term arginine supplementation on nitric oxide production, metabolism and performance in intermittent exercise in athletes J NutrBiochem 2009 20 6 462 468 10.1016/j.jnutbio.2008.05.005 18708287

[B23] Koppo K Taes YE Pottier A Boone J Bouckaert J Derave W Dietary arginine supplementation speeds pulmonary VO2 kinetics during cycle exercise Med Sci Sports Exerc 2009 41 8 1626 1632 10.1249/MSS.0b013e31819d81b6 19568197

[B24] Tang JE Lysecki PJ Manolakos JJ MacDonald MJ Tarnopolsky MA Phillips SM Bolus arginine supplementation affects neither muscle blood flow nor muscle protein synthesis in young men at rest or after resistance exercise J Nutr 2011 141 2 195 200 10.3945/jn.110.130138 21191143

[B25] Hickner RC Fisher JS Ehsani AA Kohrt WM Role of nitric oxide in skeletal muscle blood flow at rest and during dynamic exercise in humans Am J Physiol 1997 273 1 Pt 2 H405 H410 9249515 10.1152/ajpheart.1997.273.1.H405

[B26] Blum A Hathaway L Mincemoyer R Schenke WH Kirby M Csako G Waclawiw MA Panza JA Cannon RO Effects of oral L-arginine on endothelium-dependent vasodilation and markers of inflammation in healthy postmenopausal women J Am CollCardiol 2000 35 2 271 276 10.1016/S0735-1097(99)00553-7 10676669

[B27] Evans RW Fernstrom JD Thompson J Morris SM Kuller LH Biochemical responses of healthy subjects during dietary supplementation with L-arginine J Nutr Biochem 2004 15 9 534 539 10.1016/j.jnutbio.2004.03.005 15350985

[B28] Schwedhelm E Maas R Freese R Jung D Lukacs Z Jambrecina A Spickler W Schulze F Böger RH Pharmacokinetic and pharmacodynamic properties of oral L-citrulline and L-arginine: impact on nitric oxide metabolism Br J Clin Pharmacol 2008 65 1 51 59 10.1111/j.1365-2125.2007.02990.x 17662090 PMC2291275

[B29] Sureda A Cordova A Ferrer MD Effects of L-citrulline oral supplementation on polymorphonuclear neutrophils oxidative burst and nitric oxide production after exercise Free Radic Res 2009 43 9 828 835 10.1080/10715760903071664 19585317

[B30] Koifman B Wollman Y Bogomolny N Chernichowsky T Finkelstein A Peer G Scherez J Blum M Laniado S Iaina A Keren G Improvement of cardiac performance by intravenous infusion of L-arginine in patients with moderate congestive heart failure J Am Coll Cardiol 1995 26 5 1251 1256 10.1016/0735-1097(95)00318-5 7594039

[B31] Böger RH Bode-Böger SM Thiele W Creutzig A Alexander K Frölich JC Restoring vascular nitric oxide formation by L-arginine improves the symptoms of intermittent claudication in patients with peripheral arterial occlusive disease J Am Coll Cardiol 1998 32 5 1336 1344 10.1016/S0735-1097(98)00375-1 9809945

[B32] Piatti P Fragasso G Monti LD Setola E Lucotti P Fermo I Paroni R Galluccio E Pozza G Chierchia S Margonato A Acute intravenous L-arginine infusion decreases endothelin-1 levels and improves endothelial function in patients with angina pectoris and normal coronary arteriograms: correlation with asymmetric dimethylarginine levels Circulation 2003 107 3 429 436 10.1161/01.CIR.0000046489.24563.79 12551867

[B33] Lucotti P Setola E Monti LD Galluccio E Costa S Sandoli EP Fermo I Rabaiotti G Gatti R Piatti P Beneficial effects of a long-term oral L-arginine treatment added to a hypocaloric diet and exercise training program in obese, insulin-resistant type 2 diabetic patients Am J Physiol Endocrinol Metab 2006 291 5 E906 E912 10.1152/ajpendo.00002.2006 16772327

[B34] Böger RH Bode-Böger SM Szuba A Tsao PS Chan JR Tangphao O Blaschke TF Cooke JP Asymmetric dimethylarginine (ADMA): a novel risk factor for endothelial dysfunction: its role in hypercholesterolemia Circulation 1998 98 18 1842 1847 10.1161/01.CIR.98.18.1842 9799202

[B35] Tsikas D Böger RH Sandmann J Bode-Böger SM Frölich JC Endogenous nitric oxide synthase inhibitors are responsible for the L-arginine paradox FEBS Lett 2000 478 1–2 1 3 10922458 10.1016/s0014-5793(00)01686-0

[B36] Hov GG Sagen E Bigonah A Asberg A Health-associated reference values for arginine, asymmetric dimethylarginine (ADMA) and symmetric dimethylarginine (SDMA) measured with high-performance liquid chromatography Scand J Clin Lab Invest 2007 67 8 868 876 10.1080/00365510701429836 17852822

[B37] Teerlink T Measurement of asymmetric dimethylarginine in plasma: methodological considerations and clinical relevance Clin Chem Lab Med 2005 43 10 1130 1138 16197310 10.1515/CCLM.2005.197

[B38] Horowitz JD Heresztyn T An overview of plasma concentrations of asymmetric dimethylarginine (ADMA) in health and disease and in clinical studies: methodological considerations J Chromatogr B Analyt Technol Biomed Life Sci 2007 851 1–2 42 50 10.1016/j.jchromb.2006.09.023 17045556

